# Telehealth as a Bright Spot of the COVID-19 Pandemic: Recommendations From the Virtual Frontlines ("Frontweb")

**DOI:** 10.2196/19045

**Published:** 2020-06-25

**Authors:** J Nwando Olayiwola, Candy Magaña, Ashley Harmon, Shalina Nair, Erica Esposito, Christine Harsh, L Arick Forrest, Randy Wexler

**Affiliations:** 1 Department of Family Medicine The Ohio State University Wexner Medical Center Columbus, OH United States; 2 The Ohio State University Wexner Medical Center Columbus, OH United States; 3 Department of Otolaryngology The Ohio State University Wexner Medical Center Columbus, OH United States

**Keywords:** telehealth, telemedicine, primary care, COVID-19, pandemic, outbreak, public health, infectious disease

## Abstract

The coronavirus disease (COVID-19) pandemic has accelerated the telehealth tipping point in the practice of family medicine and primary care in the United States, making telehealth not just a novel approach to care but also a necessary one for public health safety. Social distancing requirements and stay-at-home orders have shifted patient care from face-to-face consultations in primary care offices to virtual care from clinicians’ homes or offices, moving to a new frontline, which we call the “frontweb.” Our telehealth workgroup employed the Clinical Transformation in Technology implementation framework to accelerate telehealth expansion and to develop a consensus document for clinician recommendations in providing remote virtual care during the pandemic. In a few weeks, telehealth went from under 5% of patient visits to almost 93%, while maintaining high levels of patient satisfaction. In this paper, we share clinician recommendations and guidance gleaned from this transition to the frontweb and offer a systematic approach for ensuring “webside” success.

## Introduction

If I knew I could see you this way, I would have done this a long time ago. I don’t wanna come to your office with my baby and put him at risk for Corona.

Family Medicine telehealth patient

Six months ago, the Ohio State University Wexner Medical Center launched an all-encompassing virtual health initiative for primary care clinicians (in the Departments of Family Medicine and General Internal Medicine) and their teams, allowing them to provide telehealth to their patients via a variety of modalities and options. For Family Medicine, this meant delivering virtual health to patients in 9 primary care locations across Central Ohio. This expanded the health reach of Wexner Medical Center’s previous offerings in various telehealth initiatives that had been suboptimal but which were ready for expansion. With a new department chair as a passionate telehealth proponent and a new chancellor that articulated a visionary blueprint for the adoption of virtual care, the department was highly motivated, setting a goal of transitioning 30% or more of its routine primary care visits for over 90,000 patients to virtual visits over the next 3-5 years. The department formed a telehealth workgroup within its new Center for Primary Care Innovation and Transformation, and this team applied accepted principles for technology integration in primary care [1] to achieve this goal. At this time, it was known that at least 42% of hospital systems and medical centers had some sort of telehealth capability [2], but adoption had generally been limited by payor reimbursement, regulatory and licensing policy, geography, and institutional readiness [2]. It was also understood that despite the benefits of telehealth in improving access, quality, efficiency, and cost of care [3], telehealth innovation in primary care has often failed for a number of other reasons as well, including lack of dedicated project management, limited patient engagement and support, and insufficient training [4].

In this initiative, we defined virtual health as any form of health care delivered without the patient and the clinician being present in the same physical location, and telehealth as the various digital communication modalities and applications that empower care to be delivered irrespective of space and time. Broadly, this includes remote monitoring, store-and-forward technology, mobile health applications, and direct patient care.

Primary care clinicians (PCCs) were trained in the use of four modalities of telehealth care, including:

- eVisits: electronic visits between the clinician and the patient, initiated by the patient through a patient portal for select complaints
- tVisits: telephone visits between the clinician and the patient, scheduled by the practice team or initiated by the patient, with documentation in the patient’s record
- vVisits: video visits between the clinician and the patient, scheduled by the practice team and conducted securely through an integrated video platform with documentation in the patient’s record
- eConsults: electronic consults between the primary care clinician and a subspecialist-clinician that allow clinician-to-clinician communication for specialty care–related consultation

Initially, adoption was primarily by self-selected or chair-appointed champions. Uptake was slow for myriad reasons—less than 5% of patient visits were conducted through telehealth in early 2020. The workgroup focused on removing known and identified barriers to widespread uptake and focused on a phased approach to training and securing buy-in from the clinician workforce.

## The COVID-19 Pandemic

On March 9, 2020, the first cases of coronavirus disease (COVID-19) were reported in Ohio and a state of emergency was immediately declared by the Governor. The first diagnoses of community spread in Ohio were reported on March 11, 2020, and by the end of that week, K-12 schools were closed across the state; medical centers, COVID-19 call centers, and swabbing stations were opened. The community and health care landscape had changed dramatically in just 7 days. The following week, our department enacted swift measures to convert as much nonemergent care to telehealth care for patients across the region, working to drastically reduce the volume of patients at-risk in busy waiting rooms to create a safer environment for employees, clinicians, patients, and communities. In a matter of days, we experienced a significant surge in requests by our PCCs to have medical center–issued laptops, video capability, secure telephone tools, resources for telehealth and training, office and home connectivity, and more. PCCs who were reluctant to embrace telehealth were now actively seeking it as a solution. Across the nation, many other health care systems needed to do the same.

As social distancing became increasingly paramount, the Governor closed all restaurants and bars, then public centers, gyms, movie theatres, and more on March 15 and 16, respectively. Within a few days, we rescheduled nonurgent care appointments scheduled for the rest of April to later dates and asked our medical assistants to call patients from every practice to reschedule their visits and check in on their well-being, while also inquiring about their readiness to receive care via telehealth. In the initial calls with approximately 400 patients, our patients expressed that this mode of health care delivery was new for them and posed a number of questions regarding technical requirements, virtual visit preparation, and what would be covered in these visits. Nevertheless, most were open and willing to engage in virtual care for their safety and that of their families, especially since some in-person care would still be provided as needed. However, to be able to deliver telehealth to the majority of patients and minimize the fiscal impact to the practice, there remained significant financial and regulatory barriers to overcome. On March 17, a number of major telehealth regulatory changes in response to the COVID-19 pandemic addressed the most substantial roadblocks to telehealth acceleration and adoption, including the Centers for Medicare and Medicaid broadening access to telehealth by authorizing reimbursement for video visits as well as increased flexibility around state licensure requirements for Medicare patients [5]. In addition, the Office for Civil Rights relaxed strict HIPAA (Health Insurance Portability and Accountability Act) rules around telehealth vendors, covered health care providers, location of service, and modality of communication [6]. On March 20, the American Board of Family Medicine issued an emergency decision to allow telehealth visits for resident physicians to count toward their required graduation ambulatory visit targets [7]. We rapidly scaled training for PCCs, including our resident physicians and their attending faculty, to become proficient in the delivery of virtual care, as well as guidance on important “webside” manners between patients at home and remotely stationed clinicians. Almost overnight, the increased use of telehealth became a bright spot of the pandemic [8].

## Approach

### Development of Recommendations

An increasing number of Ohioans affected by COVID-19 resulted in the need for enhanced social distancing and containment. Our department transitioned nearly all of its clinical employees to remote operations by March 23, 2020. It was quickly recognized that PCCs and clinical support staff needed detailed guidance as they moved from numerous clinical practice settings to the “frontlines,” or as we call it, the “frontweb” of the COVID-19 pandemic. In this process, numerous questions, worries, challenges, and opportunities were expressed by PCCs moving to the frontweb of this pandemic.

It was essential that the PCCs received clear and direct recommendations to optimize newer care delivery models, so the workgroup convened a multidisciplinary subset of experts to draw consensus on appropriate guidance. This team consisted of physicians, electronic health record (EHR) and information technology (IT) professionals, process improvement and ambulatory leadership, communications experts, and patient experience advocates. The group subsequently developed a concise set of recommended practices, specifically focusing on telephone and video visits toward which the bulk of our care was shifting. These recommendations include: understanding evolving federal, state, and institutional guidelines, as these change in response to the pandemic; seeking additional necessary environment training and experiential learning or various modalities and platforms; creating an ideal virtual office space and testing the technology in advance; communicating with patients about the changes while also planning to accommodate their language, disability, technical, and literacy needs; bringing a thoughtful webside manner to the visits; and suggestions for obtaining additional assistance related to technology, specialty care, personal emotional health, or complex patient needs. A summary of these recommendations can be found in Multimedia Appendix 1.

### Applying an Implementation Framework

To ensure the scalability and sustainability of these rapidly emerging changes, the workgroup applied the Clinical Transformation in Technology (CTT) implementation science-based framework, which is an established 5-component change model that enables primary care settings to be successful in technology adoption, implementation, or expansion [1]. The 5 components, or 5L phases, include *Logistics*, *Landscaping*, *Looping* (feedback), *Launching*, and *Leading and Leveraging* (learnings). Without the luxury of extended time, activities in these different components were enacted in synchrony during the pandemic response.

The following sections highlight some of the key actions, activities, barriers, and solutions in each of the 5L phases.

#### Logistics

The *Logistics* phase of the CTT framework involves legal, technical, security, identifying, anticipating, and mitigating roadblocks, as well as privacy considerations and preparation. Important activities completed by our medical center and department’s telehealth workgroups during this phase included: vetting a host of telehealth platforms and third-party solutions that could be supported, in addition to those offered in our EHR system, as well as securing the necessary agreements and support to utilize them; creating a strategy for deployment of software and hardware to the PCCs and other members of the care team; devising solutions for integration of third-party solutions with our EHR system; and ensuring ongoing HIPAA protections with all technical solutions.

#### Landscaping

The *Landscaping* phase of the CTT framework involves understanding and improving clinical, human resource or process gaps, existing workflows, and systems and setting–specific, actionable goals for success. Important activities that were undertaken to re-engineer processes and workflows during this phase included: revising schedule templates and expanding visit type architecture; creating specific scheduling workflows for various clinician and care team member roles; ensuring device and software compatibility with various telehealth platforms; developing virtual patient check-in and check-out procedures; updating billing protocols, including modifiers, CPT (Current Procedural Terminology) codes and time documentation; building in-person care teams and articulating appropriate clinical conditions for patients requiring a physical visit; setting agreed upon SMART (Specific, Measurable, Actionable/Achievable, Relevant/Realistic and Time-Bound) goals and timelines; and consistently aligning patient communication and support to assist patients with the transition and technology.

#### Looping

As technical, security, workflow, clinical, communication, training, and process activities occurred, the *Looping* phase of the CTT framework became critical. In this phase, learnings, feedback, and the results of previous changes are used to drive and inform improvement and iteration. Applying rapid-cycle improvements in real time significantly accelerated our ability to scale.

#### Launching and Leading

The *Launching and Leading* phase involves adequate training, retraining, and structuring telehealth initiatives for optimal impact. Because our clinicians had been previously trained in most telehealth modalities, we were able to provide focused training online to fill in gaps, refresh previous training, and create practical tip sheets. Important activities during this phase included the establishment of regular, frequent department telehealth workgroup office hours to allow clinicians and staff to be able to troubleshoot issues in real time; optimization of patient communication materials; advancing the patient scheduling process to accommodate patient visit modality preferences; revisiting appropriate documentation workflows and virtual visit amenable complaints or conditions; applying protocols for home-based monitoring and patient-initiated reporting; and streamlining the use of internal and third-party telehealth solutions.

#### Leveraging

Finally, in the *Leveraging* phase, all previous learnings are used to drive scale and spread. Activities we have embarked on during this phase include the development of performance and operational dashboards that align with SMART goals; creating a mechanism through which actionable department-, practice-, and clinician-level data are monitored and shared on a weekly basis with department and clinical leadership; and ensuring that patient preference, experience, comfort, and well-being are systematically assessed.

## Results

As PCCs moved from the primary care office to the frontweb of the pandemic, we provided over 1500 telehealth visits within the first few days (Table 1). Over the following few weeks, we experienced a considerable increase in telehealth engagement, with nearly 93% of current care being delivered through telehealth/virtual care, while the ongoing provision of in-person care when it is necessary or preferred by patients continued (Table 1). In the same period, patient satisfaction has remained at prepandemic high levels, per very preliminary internal patient experience data.

With this level of acceleration, it became imperative that our telehealth workgroup continue leveraging learnings and looping feedback as part of the CTT framework. The workgroup continues to actively work to revise and improve processes and workflows, based on patient technical and connectivity challenges, clinician feedback and technical questions, software and third-party solution differences, state and federal guidance, and institutional recommendations and algorithms. The majority of primary care video visits has been conducted through a platform called Updox, while additional video visits have been completed through Epic MyChart and Doximity. Options for using FaceTime, Skype, and Zoom have also been made available.

**Table 1:** Summary of COVID-19 telehealth visit acceleration in the Department of Family Medicine, Ohio State University Wexner Medical Center (the denominator for all percentages is defined as the number of total visits [in person + video + telephone]).

Week^a,b^	Visits, N	Telehealth visits^c^, n (%)	In-person visits, n (%)	Video visits, n (%)	Telephone visits, n (%)
03/01/20	2822	4 (0.1)	2818 (99.9)	4 (0.1)	0 (0)
03/29/20	1814	1666 (91.8)	148 (8.2)	386 (21.3)	1280 (70.5)
04/26/20	2104	1947 (92.5)	157 (7.5)	1481 (70.4)	466 (22.1)

^a^Data shown here represent 4-week intervals.

^b^Week 03/01 represents pre-COVID operations data. Week 3/29 represents the official launch of video visits across the entire medical center. Weeks 03/01 to 03/29 represents a shift from majority in-person visits to majority virtual telehealth visits overall (phone and video). Weeks 03/29 to 04/26 represents a shift from majority phone visits to majority video visits for all virtual telehealth visits overall.

^c^Telehealth visits include patient visits conducted by Family Medicine physicians and nurse practitioners through tVisits or vVisits (telephone or video); does not include data from Behavioral Health, Clinical Pharmacy, Nutrition or other clinicians.

Telehealth care has been applied for a wide range of primary care needs, such as chronic disease management, well-person care (encompassing physical exams and well-child visits) and wellness checks, mental health follow-up, medication management, new patient encounters, acute nonemergent complaints such as back pain, headache, and rash, and lifestyle counseling. Options for subspecialty consultation, diagnostic testing (lab or radiology), and urgent and emergent care remain available.

## Conclusion

Academic medical centers and health systems across the nation have done a tremendous job in responding to an unprecedented pandemic with a panoply of tools to provide high-quality clinical care while keeping their employees, patients, and communities as safe as possible [8]. Rapid adoption or expansion of telehealth care has become one of the central components of the pandemic response [8-10]. As the nation continues to confront its post-COVID-19 future and anticipates its new norm, the fate of primary care telehealth will be determined by (1) which system, regulatory, financial, policy, and clinical adaptations continue to stand; (2) institutional propensity to scale and sustain efforts; and (3) the desire of patients to engage in new modalities of care [8]. We believe that PCCs will maintain some sort of permanency on the frontweb of care and that many patients will appreciate the convenience that telehealth has brought them. However, these are areas for future study. Sharing recommendations, best practices, lessons learned, and strategies to thrive in an ever-changing landscape will become core to the new norm in primary care.

## Appendix

Multimedia Appendix 1 The Primary Care Clinician Recommendation Checklist for delivering telehealth remotely.

## Abbreviations

COVID-19: coronavirus disease
CPT: Current Procedural Terminology
CTT: Clinical Transformation in Technology
EHR: electronic health record
eConsult: electronic consult
eVisit: electronic visit
HIPAA: Health Insurance Portability and Accountability Act
IT: information technology
PCC: primary care clinician
SMART: Specific, Measurable, Actionable/Achievable, Relevant/Realistic and Time-Bound
tVisit: telephone visit
vVisit: video visit
