# Supplementary material for: Telehealth as a Bright Spot of the COVID-19 Pandemic: Recommendations From the Virtual Frontlines ("Frontweb")
Source: JMIR Public Health Surveill. 2020 Jun 25;6(2):e19045. doi: 10.2196/19045 (PMC7318864; doi:10.2196/19045)
Supplement: Multimedia Appendix 1 [file publichealth_v6i2e19045_app1.pdf]

Multimedia Appendix 1.

Delivering Telehealth Remotely: The Primary Care Clinician Recommendation Checklist

Created by the Ohio State Department of Family Medicine Telehealth Workgroup | March 2020

As primary care clinicians begin adopting various telehealth modalities and moving clinical operations into their home, here are some useful recommendations we've developed with a multi-discplinary team of experts, to foster high tech, high touch and high quality patient encounters right from your own home or office, with the right "webside manner." Keep these recommendations in mind as we enter patients homes and spaces or they enter ours, on the "frontweb"or frontlines of care.

Recommendation 1: Understand Professional Society, State and Federal, and Organizational Telehealth Recommendations

- Review your institution or practice's recommendations for telehealth and understand available tools
- Understand additional guidance and policies, such as billing, reimbursement, scope of practice & documentation, from federal and state governments

Recommendation 2: Get Hands-on Training in the Telehealth Tools Offered by Your Institution or Practice

- Get the training or re-training you need and make sure the training accommodates real or mock cases you are likely to see
- Familiarize yourself with various telehealth platforms and the benefits of each
- Consider having a trainer shadow you in real time as you deliver virtual care
- Gain comfort with the telehealth platforms available to you and learn to use different modalities to be able to adapt to patient preferences and needs

Recommendation 3: Set up Your Virtual Health "Office" Space for Success

- Determine a prime location for virtual care, and organize your "virtual office" to be clutter-free, without too many personal effects in the background
- Ensure good lighting for video encounters, and minimize sound/noise disruption for video and telephone encounters
- Make sure your office is a space where only you will be present—no family members, pets, etc. should be present

Recommendation 4: Communicate with Your Patients

- Conduct patient outreach and communicate on the new modalities of care you are offering or how this may be different than previous encounters
- Set virtual office hours and inform patients of how and when to reach you
- Develop clear and patient-friendly talking points for clinical and scheduling staff to discuss with patients

- Anticipate and plan for special needs and technical or other accommodations patients may have before the visit

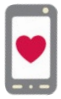

Recommendation 5: Prepare and Test Your Technology

- Conduct some practice sessions before going live with telehealth platforms
- Use the same hardware and devices in your training and practice sessions that you will use when you go live
- Utilize headphones or earbuds for improved sound quality and privacy; avoid conducting visits where others around you can hear sensitive conversations
- Check your sound, Wi-Fi and video settings before the visit

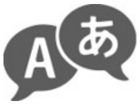

Recommendation 6: Plan for Patients With Language Interpretation or Disability Needs

- Have resources for language interpretation and disability accommodations for patients readily available
- Utilize interpretation services supported by your institution or practice
- Integrate patient accommodations into visits and post visit follow up

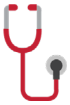

Recommendation 7: Conduct your Visit With Confidence and Great Webside Manner

- Wear professional clothing and/or white coat for video visits
- Conduct the visit securely and considering privacy needs
- Open the visit with a brief overview of what to expect and visit flow
- If pictures need to be taken or the patient needs to self-examine, explain the process in simple language
- Speak clearly and slowly so that patients can understand
- Document with additional detail on visit format, platform used and any deferred parts of the visit based on the virtual nature
- Close the visit with direct next steps and make sure the patient understands; be sure the patient has time to ask questions
- Smile! Patients can see/feel/hear your nonverbal cues and your compassion

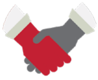

Recommendation 8: Get Help When You Need It

- Understand available resources for crisis management, behavioral health and community services
- Know where to send patients for diagnostic services, emergent or urgent care
- Be prepared with information on in-person care services if needed
- Utilize eConsults for subspecialty questions that arise if these are supported in your institution/practice
- Seek additional technical training from your IT or training teams as needed
